# Bilateral single-system ectopic ureters opening into vaginalized urogenital sinus

**DOI:** 10.4103/0970-1591.60460

**Published:** 2010

**Authors:** Bhupendra P. Singh, Hemant R. Pathak, Mukund G. Andankar

**Affiliations:** Department of Urology, BYL Nair Charitable Hospital and Topiwala National Medical College, Mumbai Central, Mumbai - 400 008, India

**Keywords:** Ectopic ureter, ureter, urogenital sinus

## Abstract

A 5-year-old female presented with continuous dribbling of urine without any voiding stream since birth. Upon investigations, the bladder neck and both ureters were opening into the vaginalized urogenital sinus and the urethra was absent. Coarctation of the aorta was an associated anomaly. To our knowledge, this is the first report in literature of bilateral single-system ectopic ureters opening into vaginalized urogenital sinus. The report highlights the necessity for consideration of continent diversion in such cases because of the absence of the urethra in addition to an incontinent bladder neck and tiny dysfunctional bladder.

## INTRODUCTION

Although 20% of ectopic ureters are bilateral, bilateral single-system ectopic ureters are rare. Urogenital sinus malformations are classified as high and low with wide and narrow groups in each class. When the vaginal introitus is near normal with the urethra inserting into a well-developed vagina, it is called vaginalized urogenital sinus. We report the first case of bilateral single-system ectopic ureters opening into vaginalized urogenital sinus.

In our case, the bladder neck and both ureters were opening into vaginalized urogenital sinus and the urethra was absent. Bilateral single-system ectopic ureters opening into urogenital sinus present a unique challenge for genitourinary reconstruction because of the absence of the urethra in addition to an incompetent vesical neck and the small capacity of the bladder.

## CASE REPORT

**Figure 1:**
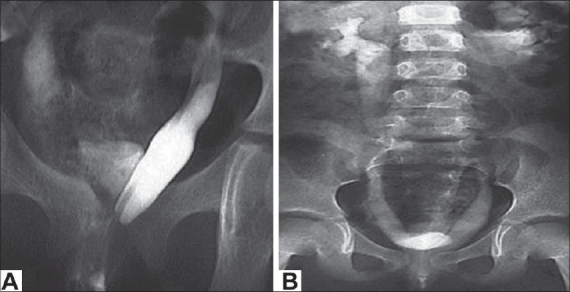
(A) A retrograde contrast study showing reflux into the right ureter and hypoplastic bladder; (B) IVU: Bilateral hydronephrosis, hydroureter, and small capacity bladder

**Figure 2:**
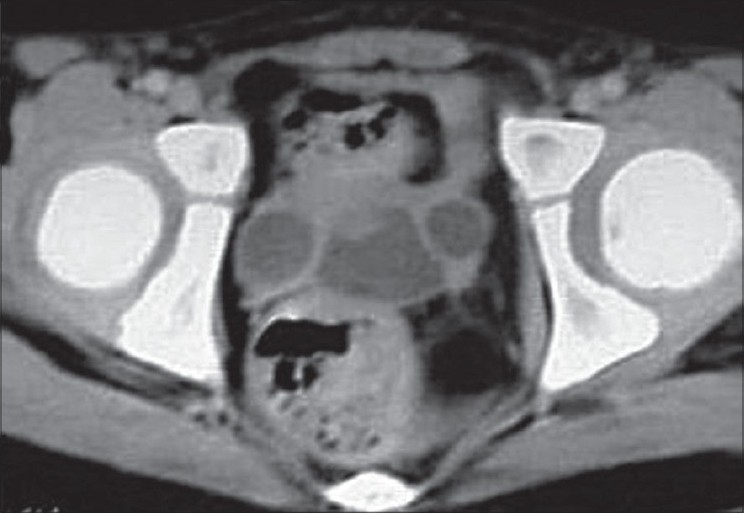
Computed tomography scan: Bilaterally dilated ureters with a small bladder

**Figure 3:**
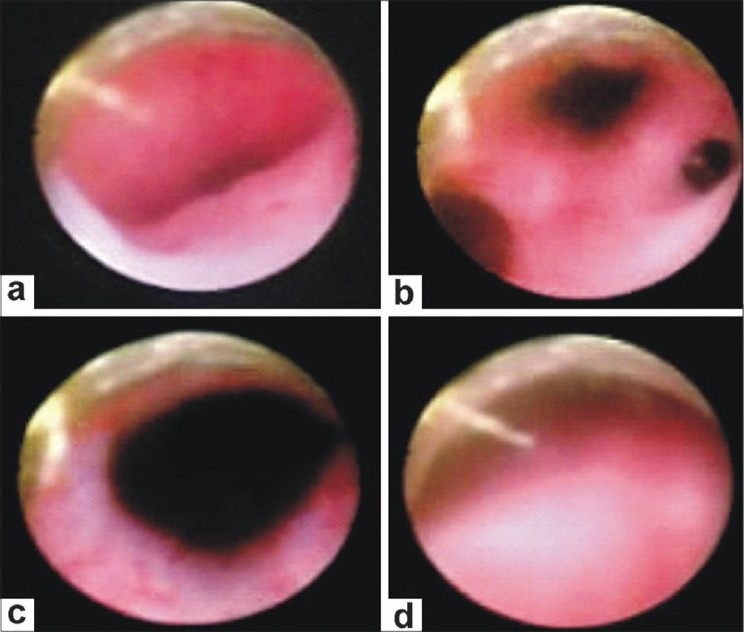
Cystoscpy (A) Urogenital sinus; (B) Bladder neck with bilateral dilated ureteric orifices opening in urogenital sinus; (C) Grossly incompetent bladder neck; (D) Poorly developed trigone

A 5-year-old female presented with continuous dribbling of urine without any voiding stream since birth. Because of poor socioeconomic status and an illiterate rural background, the parents consulted a specialist only when the child reached a school-going age. There was no history of any bowel complaints. On general examination, her blood pressure was 140/74 mmHg in the left forearm. There were no skeletal defects, anal tone and neurological examination were normal. The external genitalia was of the female phenotype and on separating the labia majora, a single opening with a continuous urine leak was seen. All routine investigations such as hemoglobin, total leucocyte count, renal function tests, and urine microscopic examination were within the normal range. On abdominal sonography, bilateral moderate hydronephrosis and hydroureters were seen. Renal parenchymal thickness, echogenicity, and corticomedullary differentiation were normal on both sides. The uterus and fallopian tubes were normal but the ovaries could not be seen. On micturating cystourethrogram catheter went into the left ureter, which was dilated; however, there was contrast overspill into the bladder and reflux was seen into the right ureter, which was also dilated [Figure 1A]. An intravenous pyelography showed good contrast excretion on both sides and dilated ureters were seen up to the lower part of the bladder, which was faintly visualized with the contrast [Figure 1B]. A computed tomography (CT) scan showed a low capacity bladder with urogenital sinus [Figure 2]. On urogenital sinus scopy and cystoscopy [Figure 3], both dilated ureteric orifices were found to open into vaginalized urogenital sinus by the side of the bladder neck. The bladder neck was wide open and incompetent. The bladder had a smooth lining without identifiable trigone. The cystometric capacity was 20 cc. Karyotype by fluorescence *in-situ* hybridization was 46 XX. Because of high blood pressure, two dimensional echocardiography was done that revealed coarctation of the aorta distal to the left subclavian artery.

We plan for continent diversion with cutaneous stoma in this case once the patient undergoes surgery for coarctation of the aorta.

## DISCUSSION

We report a case of bilateral single-system ectopic ureters opening into urogenital sinus associated with the absence of the urethra, hypoplastic bladder, incontinent vesical neck, and bilateral ureteric reflux. This is a highly unusual location for bilateral single-system ectopic ureters. There is only one other report of bilateral single-system ectopic ureters opening into urogenital sinus by Sheldon and Welch.[1] In their case, urogenital anomalies were more severe i.e., the vagina was rudimentary and both kidneys were dysplastic, resulting in end-stage renal disease. In our case, urogenital sinus was vagina-like i.e., vaginalized and both kidneys were normal. Coarctation of the aorta was an associated anomaly.

This re-emphasizes the need for continent diversion as the primary procedure in complex cases of bilateral single-system ectopic ureters with the absence of trigone and the urethra along with incompetent bladder neck and hypoplastic bladder. Kesavan, *et al*.,[2] showed the bladder neck and trigone to be maldeveloped in 75% of bilateral and 54% of unilateral ectopic ureters. Moreover, separating these refluxing ureters from the bladder neck might further damage the already incompetent neck. Furthermore, our patient is 5 years old and her bladder, inspite of a refluxing vesical neck, has a capacity of only 20 cc, which is evidence in favor of a nonfunctional and noncompliant bladder. Hence, bladder neck preservation and enhancing the bladder capacity by ureteric re-implantation or side wall ureterovesicostomy are not viable options in this case. Although bladder preservation and bladder neck reconstruction as well as urethral reconstruction have been advocated by many in cases of bilateral single-system ectopic ureters or solitary ectopic ureter, in these cases ectopic ureters opened either into a normal urethra or vagina with a normal urethra. In the only similar reported case in literature, Sheldon performed the continent diversion by orthotopic gastric neobladder with an appendix implanted in a Mitrofanoff fashion and brought out as orthotopic neourethra anterior to the vagina. In a series by Wakhlu, *et al*.,[3] three out of four females who underwent ureteric reimplantation for bilateral single-system ectopic ureters remained incontinent. In Jayanthi's series[4] of 7 patients, the total day and night time continence was only achieved by bladder neck closure, appendicovesicostomy, and augmentation rather than a bladder outlet resistance increasing procedure. Furthermore, Heuser, *et al*.,[5] also reminded us of the insufficient development of trigone and bladder neck with subsequent urinary incontinence in bilateral single-system ectopic ureters. Although they described the use of an artificial urinary sphincter (AUS) in these cases, in a patient without a urethra, this approach may not be feasible.

Our case report adds to the spectrum of literature on bilateral single-system ectopic ureters. In our opinion, continent diversion should be adopted as a single stage definite procedure in such difficult and rare cases to avoid continuing disabilities and poor outcome in terms of upper and lower tract function. Hence, in cases of bilateral single-system ectopic ureters, management modalities should differ from patient to patient depending upon the anomalies in the lower urinary tract as well as the reproductive tract.
